# Security Analysis of Cyber-Physical Systems Using Reinforcement Learning

**DOI:** 10.3390/s23031634

**Published:** 2023-02-02

**Authors:** Mariam Ibrahim, Ruba Elhafiz

**Affiliations:** Department of Mechatronics Engineering, German Jordanian University, Amman 11180, Jordan

**Keywords:** SARSA, reinforcement learning, optimal path, cyber security

## Abstract

Future engineering systems with new capabilities that far exceed today’s levels of autonomy, functionality, usability, dependability, and cyber security are predicted to be designed and developed using cyber-physical systems (CPSs). In this paper, the security of CPSs is investigated through a case study of a smart grid by using a reinforcement learning (RL) augmented attack graph to effectively highlight the subsystems’ weaknesses. In particular, the state action reward state action (SARSA) RL technique is used, in which the agent is taken to be the attacker, and an attack graph created for the system is built to resemble the environment. SARSA uses rewards and penalties to identify the worst-case attack scenario; with the most cumulative reward, an attacker may carry out the most harm to the system with the fewest available actions. Results showed successfully the worst-case attack scenario with a total reward of 26.9 and identified the most severely damaged subsystems.

## 1. Introduction

Cyber-physical Systems (CPSs) have emerged as a result of integrated progress in information technology. A CPS is a contemporary control system that incorporates a computer, communication, and control technology into physical systems. It monitors and regulates physical processes in real time using a computational system [1].

Currently, CPS has a wide range of potential applications as one of the key technologies in industry 4.0, including aerospace, civil infrastructure energy, intelligent manufacturing, intelligent transportation, etc. The widespread use of wireless networks in CPS has improved lifestyle and output, but security issues have also been introduced. Through a variety of assault techniques, cyber attackers can disable the system’s physical control mechanism. Recent CPS security breaches have shown that it is extremely important to investigate the security issue of CPS under cyber assaults [2].

The security of CPS was studied through our earlier work, such as [3,4,5], where we sought to visualize the attack sequence within the attack graph that an attacker may carry out to compromise the system. Additionally, a variety of frameworks were employed in these studies, including the use of architecture analysis and design language for system modeling, matrices, and algebra to determine any assault that may occur, and Q-based reinforcement learning (RL) for establishing the worst damage an attacker can cause.

Machine learning (ML) is a developing technology that can help with practical communication issues in actual contexts. Artificial intelligence (AI) technology is currently playing a significant part in the development of several fields. Our main contribution in this paper is to present a security analysis framework built on RL. In particular, the state action reward state action (SARSA) approach is employed which uses rewards and penalties to interact with sensory information and the environment. In contrast to previous ML techniques that rely on trials and failures, RL agents may learn on their own without prior knowledge of the environment [6].

The novelty of this work lies in introducing a unique SARSA-based on-policy algorithm to discover the ideal pathway/attack scenario in the attack graph an attacker can pursue to cause the maximum damage to CPSs through a case study of a smart grid. An off-policy learner gains knowledge of the value of the best course of action apart from the actions of the agent. An agent learning on-policy gains knowledge of the policy’s benefits, including the investigation procedures [7]. Furthermore, this approach can be useful in detecting the most susceptible section of the system, which can aid in the development of a more secure system. This strategy is compared with earlier work [5] based on Q-learning (off-policy) to discover the differences among various RL approaches.

Despite several prior studies applying the SARSA to identify the optimum path, this is the first time the SARSA algorithm is used to determine the best course of action for the attacker, assuming that the attacks are the agent’s activities and that the RL environment is the attack graph with the rewards’ values determined using the CVSS. This research offers a method that integrates the attack graph and SARSA to examine the cyber security of CPSs through a case study of a smart grid.

The rest of the paper is organized as follows: Section 1.1 highlights some of the research that has been conducted in this area. Section 2 contains the specifics of our method. The SARSA algorithm process is illustrated in Section 3 while the system’s evaluation and outcomes are provided in Section 4. Finally, we summarize our contribution and forthcoming initiatives in Section 5.

### 1.1. Related Work

Numerous works have focused on ML and RL, including [8], where the author used the evolution strategy (ES) in the training of a neural network controller for the task of pendulum control. The trials’ findings demonstrated that ES could successfully train the multi-layer perceptron (MLP) to quickly make the pendulum upright if the MLP had a sufficient number of hidden units. For the task utilized in this study, 32 hidden units did not substantially outperform 16 hidden units, while 8 hidden units performed noticeably worse than both 16 and 32 hidden units. A total of 16 units outperformed the other 2 options in terms of task performance and computing effectiveness. Additionally, the findings showed that exploration rather than exploitation helps ES search for better solutions. To determine if this conclusion holds for evolutionary algorithms other than ES, more analyses are necessary. Additionally, the study’s evolutionary algorithms should be enhanced and further tested by using them for RL tasks other than pendulum control. 

A rapid algorithm is suggested by [9] for a game player that can produce results quickly. However, there are still many optimizations to be conducted, and the objective of producing a generic game player needs further investigation. The typical player of games can currently recognize the game setting, game actor, movements, and goal state.

To provide patients with individualized therapy, the researcher in [10] developed an easy user interface for clinicians to utilize at the patient’s bedside to forecast prostate cancer survival. With a margin of error of two months, the neural network was able to predict the survival of patients with prostate cancer using the inputs of age, race, comorbidities, and baseline prostate-specific antigen (PSA). The error margin of the neural network was almost 37.5 times less than the margin of the linear regression model, which had a margin of 75 months. The best neural network model included a stochastic gradient descent optimizer, an early stopping time of 50 iterations, a minimal delta of 0.0001, and a mean squared error monitor, which produced the final margin.

Many pieces of research were conducted to explore SARSA algorithms to determine the optimal path, such as [11], which offers a unique RL method called “Iterative SARSA” that allows an agent to choose the best and safest path in dangerous surroundings filled with hazards. The suggested method works best in situations when the operations’ overall safety and efficacy take precedence over their ability to be implemented quickly. The safest trajectory is then determined using SARSA. However, the length of the path results in a rise in time complexity due to various iterations.

In order to determine the optimal path for mobile robots with the least amount of computation, a hierarchical path planning technique using multi-SARSA based on topological maps is proposed in [12]. The approach put forward in this research splits and resolves the issue by supplying a priori knowledge: the topological map gives the initialization Q-table, while the artificial potential field technique supplies the child nodes. Two preceding sets of data working together make it possible to speed up the algorithm’s convergence and deliver a better outcome. The experiment investigated the impact of prior knowledge on RL path planning further. Experiments revealed that the presentation information may accelerate the algorithm’s convergence speed, and the convergence result is more comparable to the presentation information, indicating that the suggested technique has a promising future in personalized route training. The constraint, however, is in the interactive alternatives that the robot must go through. 

The potential uses of SARSA in cyber security are also investigated in the literature. For instance, Ref. [13] introduced adaptive RL methods to examine the secure state estimation problem from three distinct angles. The algorithms may determine the matching best course of action from the viewpoint of a sensor or an attacker, as well as the Nash equilibrium course of action from the viewpoint of a zero-sum game. The simulation results showed that two different kinds of algorithms could provide the best laws. It should be mentioned that there are limitations since Q-learning and SARSA rely on tables to store value; they cannot be modified to fit situations involving high-dimensional state spaces.

An impersonation attack detection approach is presented by [14] using physical layer security technology and a RL algorithm is suggested to examine the impersonation problem between edge nodes and mobile users in edge computing settings. A detection approach based on the SARSA algorithm is built under the Impersonation Attack Model (IAM) in an edge environment, detecting impersonation assaults in a dynamic setting. According to the experimental findings, the SARSA-based impersonation detection system has a slightly greater miss detection rate than Q-learning, but a lower false alarm rate and average error rate. In an edge computing environment with increased precision, communication security is therefore better safeguarded between edge nodes and mobile users.

The development of intelligent technologies in smart power systems has made various cyber physical power system architectures more vulnerable to cyberattacks. The functioning of the smart power grids can be improved in terms of efficiency and failure avoidance via the application of intelligent approaches based on ML techniques. The network can be more robust if its users are aware of the various cyberattack techniques that may be used against power systems and how to counter them [15].

Blockchain applications for the smart grid are examined in article [16], including distributed ledger technology, peer-to-peer (P2P) commerce, transactions, cyber security, and local energy markets. The authors started by meticulously going through the blockchain’s past. The smart contract and its significance in the blockchain were also explained. Finally, they discussed some of the difficulties which encounter blockchain applications for the smart grid.

A deep Q-Learning-based false data injection attack generator is developed in [17] using several probable attack scenarios. A snapshot ensemble deep neural network and a deep auto-encoder were also used to construct a two-layer framework for attack detection that can distinguish between passive and active threats. The first layer’s accuracy was 98.02%, which indicates excellent performance. With an astonishingly low false positive rate of 2.9%, the second layer, which was in charge of threat hunting, was able to detect unidentified attacks. In the end, the GridLAB-D, ns-3, and Framework for Network Co-Simulation (FNCS) simulators were combined to simulate the proposed attack modeling and detection system.

## 2. Preliminaries

### 2.1. Case Study

This section discusses the case study, which will focus on the smart grid system (as referred to in our previous work [4]). A CPS that frequently faces technological pressures is the smart grid. The term “smart grid” refers to an electricity network that can effectively integrate the behavior and operations of all users connected to it (consumers, producers, and those who do both), resulting in a financially viable, sustainable power system with minimal losses, high levels of efficiency, and elevated levels of security [18].

**Figure 1:**
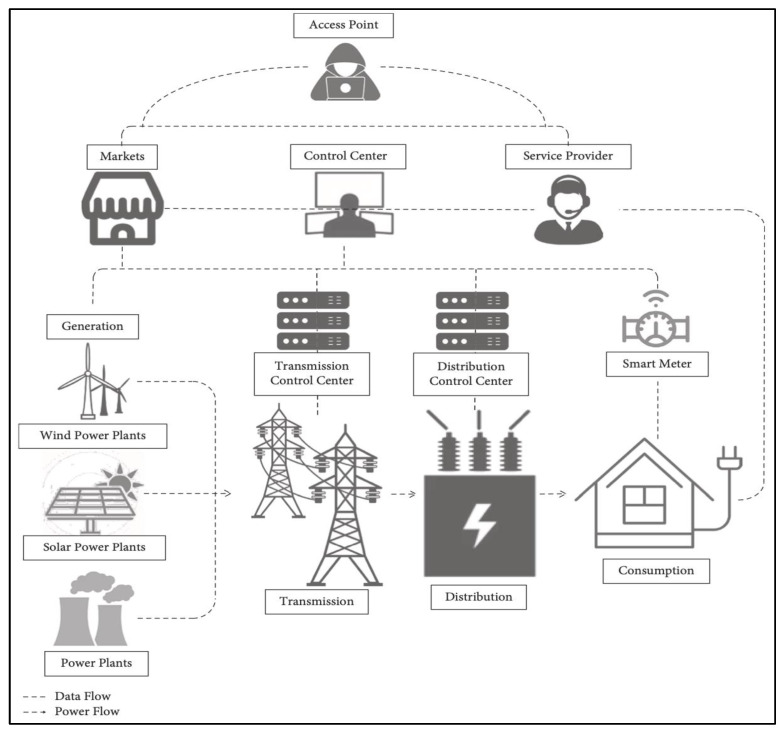
Smart grid architecture.

Figure 1 shows the components and connections of the smart grid. The generating system (GS) of a smart grid converts bulk energy into electrical energy and is connected directly to the transmission system (TS), and the transmission control center (TCC) often oversees and coordinates TS from a distance. TS transports electrical energy to be generated in further regions. It delivers electrical energy to the distribution system (DS), and a distribution control center (DCC) controls and monitors DS from a distance. Then, the DS distributes the power to the consumers (C).

This system is additionally supported by the service provider (SP), who contracts with customers to distribute power to specific devices and collaborates with internal devices via signals sent by the smart meter (SM). A broader market and system members are reached much more quickly and online by the market (MK) system, which also distributes value. The domains of the MK and SP make up the management system (M), which is in charge of managing services such as energy distribution.

The control center (CC), which understands the intelligent warning ahead by monitoring the dominating transmission network over time and assessing its security through cooperation among several specialist teams, is the last component of the smart grid. To promote a higher cognitive process, CC optimizes the transmission operation by compiling, integrating, analyzing, and mining the operational data. Additionally, it makes sure that the electrical power network is reliable, efficient, flexible, economical, environmentally friendly, and safe.

The smart grid’s communication infrastructure is also built on three different kinds of networks: the home area network (HAN), which is controlled within a small area of only a few tens of meters, the neighborhood area network (NAN), which is distributed within a wider area of some few hundred meters, and the wide area network (WAN), which is controlled within a range of tens of kilometers. Please refer to [4] for further information on the smart grid’s subsystems, network, connectivity, and vulnerabilities.

### 2.2. Attack Graph

With the use of attack graphs, several vulnerabilities may be connected to develop an intrusion. A vulnerability is a defect in a system or network that might be exploited by an attacker to carry out a successful attack. They are the preconditions for some of the attacks. They can be caused by bugs, features, or human error. Attackers will attempt to take advantage of any of these, typically combining one or more, to achieve their goal [19]. An attack graph’s description of an exploit of vulnerabilities amongst linked hosts is a change in the system state, which is reflected in security-related situations [20].

The development of attack graphs has already advanced significantly, and there are now more effective methods for doing so. A network attack model is created initially with rules (exploits) for changing the attack state by following the security criteria to automatically produce an attack graph. Exploit sequences that result in an unsafe network state are developed. These sequences may then be organized in a graph [21].

Attack graphs are complicated, though, making it challenging for people to use them effectively. Even a medium-sized network may have hundreds of potential attack points, which would be too much information for a human user to handle. Using the data from the attack graph to determine which configuration parameters should be changed in order to successfully address the security concerns found is challenging. Additional work is required to assess potential configuration changes and ensure that the best adjustments are made without a thorough understanding of the security issues that currently exist [22]. 

**Figure 2:**
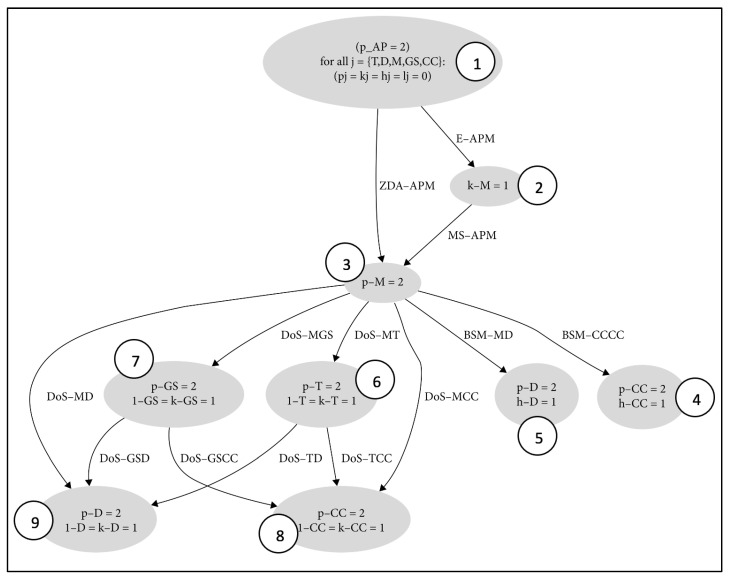
Smart grid’s attack graph.

The attack graph of the smart grid, depicted in Figure 2, will be examined in this paper. This graph was produced in our earlier work [4]. This investigation aims to further analyze the attack graph demonstrating the ease that our algorithm will provide in doing so, along with highlighting the significance of the cyber security of the smart grid.

In the smart grid, many vulnerabilities were found such as customer security (CS), which describes the large volumes of data that are autonomously collected by smart meters and sent to the market, consumers, and service providers. These data include sensitive consumer information that might be used to deduce the user’s behaviors and the devices they are using [23]. Another vulnerability in the complex system that makes up the smart grid, which is concerned with controlling both the supply and demand for power, is the greater number of intelligent devices (GNOID). These advanced gadgets might act as access points for network attacks. Furthermore, because the smart grid network is 100–1000 times larger than the Internet, network management and observation are quite challenging [24]. 

It is expected that old technology is still in use because electricity systems and relatively short-lived IT systems coexist in the same building. This equipment might serve as a weak security node and be incompatible with current power system components. This vulnerability is known as the lifetime of power systems (LOPS) [25]. A zero day (ZD) is an undisclosed computer program vulnerability that needs to be mitigated, according to those who should be concerned. Attackers can negatively exploit the vulnerability to affect programs, data, and new systems or networks until it is fixed [26].

Firmware is another weakness since it is vulnerable to many different types of software errors. These problems include memory corruption flaws, application logic errors, and command injection vulnerabilities [27].

The smart grid, which combines physical power systems with cyber systems for sensing, monitoring, communication, processing, and control, is an example of a CPS [28]. Moreover, the vulnerabilities of the smart grid can be exploited to execute cyberattacks, for example, malware spreading (MS), eavesdropping (E), denial of service (DoS), zero day (ZDA), and bypass security mechanism (BSM). Each attack instance in an attack scenario executed between two subsystems has a pre- and post-condition, in terms of connectivity, vulnerabilities, services, privileges, and attacker capabilities [4]. 

The attack graph represents the routes that the attacker can take and the attacks that can be executed to compromise the smart grid. By modifying the firmware of the DS or obtaining access to the CC, the attacker plans to disrupt the smart grid system by increasing latency in the communication between two subsystems, gaining root access on the DS, or causing a blackout.

There are sixteen sequences/paths in this attack graph that lead to states (nodes) where the system may be controlled and put in danger by the attacker. Additionally, nine nodes were located, each of which describes how the state of the system can evolve as a consequence of an attack. The system’s state captures the evolution of its dynamic variables whose values change upon attacks. These are attacker level of privilege (P), data knowledge (K), latency (L), and hardware control (H).

An example of a sequence presented in the graph can be described as follows. An Eavesdropping attack E-APM is launched on management system M in the beginning when the attacker has a privilege on the access point (AP) to obtain information (e.g., the type of smart devices and applications that M uses). In order to obtain control over M, the malware spreading MS-APM attack is then launched from the AP using the GNOID vulnerability. After that, a bypass security mechanism BSM-MD is run on D to take advantage of the LOPS vulnerability and take over the firmware of the device. This may cause a system outage and eventually have an impact on power use [4].

### 2.3. SARSA

An AI method called SARSA was built on the Markov decision process (MDP) [29]. In this work, we used SARSA to determine the optimal path of the attacker based on the rewards-augmented attack graph. The “Modified Connectionist Q-Learning MCQL” method, now regarded as one of the well-established algorithms in the RL domain of ML, was initially put out by Rummery and Niranjan [29].

Later, Sutton [30] proposed the present nomenclature SARSA. Two well-known RL algorithms (built on temporal difference (TD) learning), SARSA and Q-learning, have a great capacity for constructing a learning process that finally results in subsequent decision-making processes. The idea of an agent operating in a specific environment is used in RL to infer a policy by using a collection of self-explanatory actions, states, calculated Q-values, and reward signals.

Only by depending on its present condition and the impact of a positive or negative reward signal created by the learning process can the agent decide which action to take. Regarding the design objectives of the learning approach, a discount factor is also recommended. The impact of the upcoming reward on the present state will increase as the factor approaches unity and vice versa. Reward signals therefore act as feedback to show whether an action may succeed or fail in the learning process [31].

According to SARSA, the main method for updating the Q-value is based on the agent’s present status. The agent picks action “*a*” and the reward signal “*r*” leads the agent to choose the appropriate action; then, the agent enters state “*s*’” after executing that particular action, and lastly, the agent chooses action “*a*’” while in its new state. In SARSA, the agent conducts exploration and exploitation using the state-value function and the epsilon greedy strategy. The subsequent step assigns a grade to the states and determines their relative strength based on the weights of the derived Q-values [32]:

(1) $$V^{\pi }\left(s\right)=E\;\left[\sum_{\overset{´}{a\in A}}\gamma \;r\;\left(s,a\right)|\;s\right]$$

(2) $$Q^{\pi }\left(s,a\right)=E\;\left[\sum_{t}\gamma \;r\;\left(s,a\right)\;|\;s,a\right]$$

where:

*A*: is the agent’s actions set, $a_{i}\in A$

S: is the agent’s states set, $s_{i}\in S$

$Q_{i}\left(s,a\right):$ is the Q-value for (s,a)

$r_{i}\;\left(s,a\right)$: is the reward signal

π: is the control policy in the learning process

γ: is the discount factor

t: is a time step

Equation (1) underlines the relationship between state and value whereas Equation (2) emphasizes the association between action and value. Whether or not the agent’s present activity is known determines how significantly the two functions differ from one another. The action-value function is typically used to determine the best course of action at each time step as a result. Therefore, the following would be a better Bellman form of Equation (2):

(3) $$Q^{\pi }\left(s,a\right)=r_{i}\left(s,a\right)+\gamma \;\sum_{\overset{´}{s}\in S}P_{i}\;(\overset{´}{s}\;|\;s,a)\;Q_{i}^{\pi }\;\left(\overset{´}{s},\overset{´}{a}\right)$$

where:

$P_{i}\;(\overset{´}{s}\;|\;s,a)$: is the likelihood that an agent will change between any two successive states after performing a certain activity

$\overset{´}{s}$: is the updated state

$\overset{´}{a}$: is the updated action

The agent’s behaviors in the present and the future in SARSA are all constrained by a greedy policy and are therefore on-policy. Q-learning, on the other hand, is based on off-policy because the agent’s subsequent action is not based on the online law, prohibiting greedy behavior. The SARSA updates the Q-values using the following equation:

(4) $$Q\;\left(s,a\right)=Q\left(s,a\right)+\alpha \left(R\left(s,a\right)+\gamma Q\left(\overset{´}{s},\overset{´}{a}\right)-Q\left(s,a\right)\right)$$

where:

α: is the learning rate

R(s,a): is the reward received on moving from state s by performing an action a

$Q\left(\overset{´}{s},\overset{´}{a}\right)$: is the Q-value for $\;\left(\overset{´}{s},\overset{´}{a}\right)$

### 2.4. Common Vulnerability Scoring System (CVSS)

An open platform called CVSS is used to communicate the features and consequences of IT vulnerability. The evaluation of IT vulnerabilities, IT administrators, vulnerability bulletin providers, security organizations, application makers, and researchers will all benefit from the use of this consistent terminology [33].

The base, temporal, and environmental subcategories of the CVSS measure are separated. The inherent characteristics of a vulnerability that hold throughout time and in various user contexts are represented by the base metric category. It includes two different categories of metrics: those that evaluate the impact and those that evaluate exploitability. 

The criteria for exploitability reflect both the technical methods and the simplicity with which a vulnerability may be misused. In other words, they represent traits of the susceptible entity, also known as the vulnerable component. The impacted component, which is properly known as the object that is affected, is what the impact metrics refer to as the direct result of a successful attack [34].

The elements of a vulnerability that change over time but not across user contexts are highlighted by the temporal metric category. For instance, the CVSS score would increase if a rudimentary exploit kit was included, but it would decrease if an official remedy was developed [35].

The susceptibility traits particular to a certain user’s surroundings are represented by the environmental metric group. A system’s relative value within a technological infrastructure, the existence of security procedures that may reduce some or all of the effects of a successful assault, and other similar considerations are all important [36].

We used an online CVSS calculator [37] to determine the CVSS score for each attack on the smart grid system, as shown in Table 1. For instance, the inputs that were supplied into the calculator for the eavesdropping attack that the host AP conducted against subsystem M (E-APM) were as follows:

- Our input for the Attack Vector was local, meaning that the attack is being conducted via read/write/execute capabilities and that the vulnerable component is not connected to the network stack.
- The Low input for the Attack Complexity was entered into the calculator, signifying that no special access requirements or mitigating factors exist.
- The value entered for the Privileges Required field is None, indicating that the attacker was not authorized before beginning the attack and does not require access to the settings or data on the susceptible system to carry it out.
- None was entered in the User Interaction field, indicating that no user interaction was required to abuse the system.
- The Scope field’s response of Unchanged indicates that only resources under the control of the same security authority can be harmed by an exploited vulnerability.
- The attacker has access to some protected information, but he or she has no control over what information is gained or how much of it is obtained. This is shown by the Low response that was entered into the Confidentiality field.
- The None response was entered for the two fields Integrity and Availability, signifying that the affected component has not lost its integrity or availability.
- The input for Exploit Code Maturity is Functional, indicating that there is functional exploit code available.
- Unavailable was the response for Remediation Level and Reasonable for report Confidence.
- Security Requirements: Low
- Modified Attack Vector (MAV): Local
- Modified Attack Complexity (MAC): Low
- Modified Privileges Required (MPR): High
- Modified User Interaction (MUI): None
- Modified Scope (MS): Unchanged
- Modified Confidentiality (MC): Low
- Modified Integrity (MI): Low
- Modified Availability (MA): High
- These inputs resulted in an overall score of 3.4.

**Table 1:** Attacks’ CVSS Scores.

Attack Name	Base Score	Temporal Score	Environmental Score	Overall Score
E-APM	4.0	3.8	3.4	3.4
ZDA-APM	5.2	4.8	4.7	4.7
MS-APM	5.0	5.2	4.8	4.8
BSM-CCCC	8.0	8.1	8.1	8.1
BSM-MD	8.0	8.1	8.1	8.1
DoS-MCC	7.5	7.5	10	10
DoS-MT	9.2	9.0	8.7	8.7
DoS-MGS	9.2	9.0	8.7	8.7
DoS-TCC	7.5	7.5	10	10
DoS-TD	7.5	7.5	10	10
DoS-GSCC	7.5	7.5	10	10
DoS-GSD	7.5	7.5	10	10
DoS-MD	7.5	7.5	10	10

## 3. Methodology

**Figure 3:**
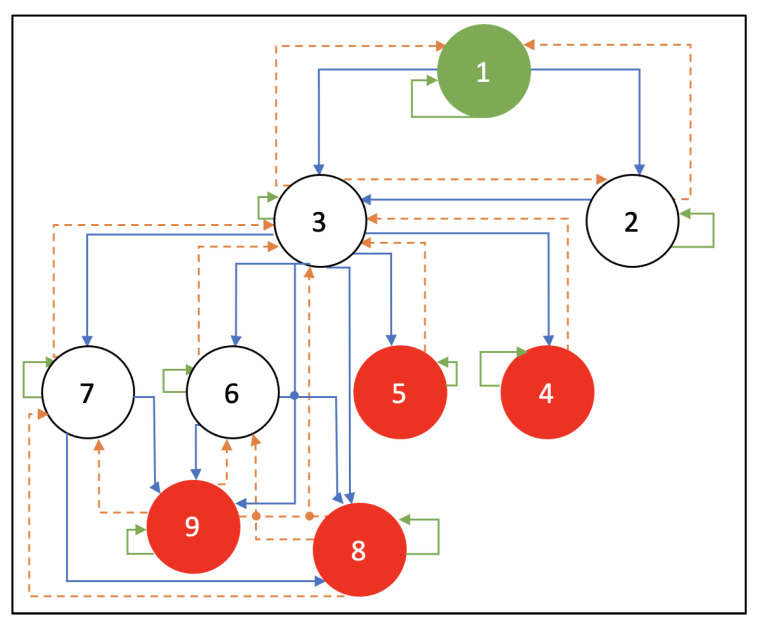
Rewards-augmented attack graph.

This section details the process of the SARSA-based rewards-augmented attack graph. The attack graph depicted in Figure 2 is duplicated to be more thorough as illustrated in Figure 3. It shows the rewards-augmented movements that the attacker can carry out from each node. The attacker’s initial location is supposed to be at node number 1, and the attacker is free to go through the nodes in any order until reaching node numbers 4, 5, 8, and 9, which represent the objective states. Additionally, the reward values are determined using the CVSS overall ratings from Table 1. Once in the target state, the attacker will remain there indefinitely. The forward route is depicted by the blue lines, and the value of these lines varies according to the attack and its CVSS value. The agent will receive no reward for any action or movement that is reversed, demonstrated using the orange lines. The reward will be -1 if the agent stays put (remains in the same node) or goes to a different node that is not linked to the one they are currently in, as illustrated with the green lines. 

The potential rewards the agent could receive when navigating between the nine nodes are shown in Table 2. For instance, the reward would be 8.1 if the attacker moved from node 3 to node 5.

Algorithm 1 displays the predicted optimal path of the attacker through the SARSA-based rewards-augmented attack graph. The initial state 1 is provided as input. However, it is presumed that nodes 4, 5, 8, and 9 are the ending states. The suggested path from the source to the destination nodes is the output. Equation (4) is used to compute and update the Q-value in the suggested algorithm for route recommendation using SARSA.

The epsilon greedy strategy is used to choose the next course of action in states. For each state, a random integer (0 or 1) is created and then contrasted with the epsilon value. This method selects the action with the lowest value for the specified user preference if the produced random number is higher than epsilon; otherwise, it takes a greedy approach to investigate all other possible actions for the specified condition. The path is forecasted taking into account the highest value of action “a” for the state “s”. Up until one of the final nodes is reached, it is repeated.
**Algorithm 1:** Predict the optimal route Input: Start state; Result: Optimal route; initialization; Initialize Q(s,a); Initialize state ’s’; Choose an action ’a’ using epsilon-greedy approach; **for**
*each time step* **do** Take a; Observe the reward r(t+1) and the state s(t+1); Update Q(s(t),a(t)); s(t) ← s(t+1); a(t) ← a(t+1) **end**

## 4. Experimental Results and Discussion

In this part, we applied our SARSA approach using Python to identify the worst-case attack scenario an attacker may carry out on the smart grid system. On a typical computer processor, the execution time for the employed approach is almost 1 h and 15 min: 2.3 GHz8-CoreIntelCorei9; memory: 16 GB, 2667 MHz DDRF4, running macOS Big Sur.

**Figure 4:**
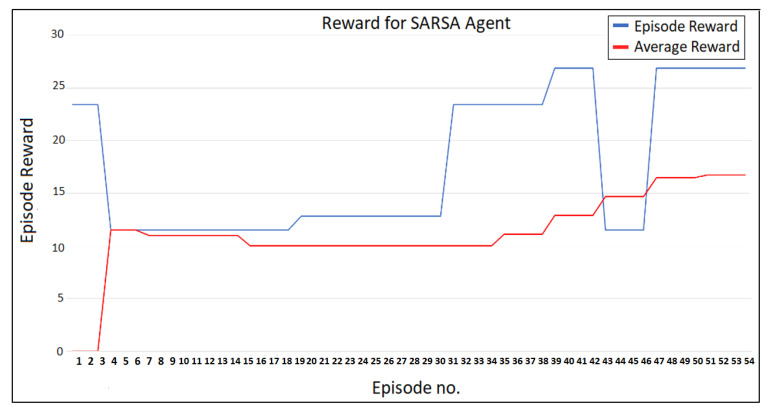
Results of SARSA agent.

Finding the optimum path for the attacker/agent reflects the agent’s training development as depicted in Figure 4. The cumulative reward for each episode is shown on the y-axis, while the x-axis shows the number of episodes. The model’s convergence required 54 episodes. The red line depicts how the average reward changed after each episode, illustrating how the agent training has changed. The blue line displays the total reward for each episode. The chart shows that the average reward is rising, indicating an improvement in the agent’s training as the episodes go.

According to the findings and after 54 iterations, the worst assault scenario path involves the nodes 1 → 2 → 3 → 6 → 9, with a total reward of 26.9. The most severely damaged subsystems that may be identified using this information are M, MT, and DS. 

The cumulative reward started low during training (when the agent was unsure of the best course of action) but increased throughout episodes as the best course of action was discovered. When the reward remained constant, the agent’s training was finished. This required 54 iterations.

To protect the vulnerable parts of the system from both internal and external assaults, network Intrusion prevention (IP) and intrusion detection (ID) technologies can be added to host-based defenses. Several ways can be addressed to safeguard the smart grid and improve its security [25], including putting in place a strong authentication mechanism, conducting yearly element vulnerability assessments, and modifying virtual private network (VPN) topologies for secure communication.

Another traditional reinforcement approach was employed in our prior work [5] to determine the best route an attacker may follow to compromise an integrated clinical environment system, and the algorithm utilized was based on Q-learning. Both Q-learning and SARSA rely on tables to store value. In [5], one target node was the lone node in the attack graph, which had seven nodes. It was found that the Q-learning results showed the shortest path, independent of the cumulative reward, as opposed to the SARSA algorithm, which disregarded the shortest path, such as 1 → 3 → 4. A longer path with greater cumulative reward was produced as a result of the SARSA. Both strategies function in a limited space (or a discretized continuous environment). While SARSA learns a near-optimal policy, Q-learning directly learns the optimal policy. Because it is optimal, the Q-learning agent will choose the shortest route, but the SARSA agent would choose the longer one with a greater cumulative reward. 

Accessibility to the system model is crucial for the growth of the attack graphs. A one-time modeling effort is frequently required to obtain the system description for components, connections, services, and vulnerabilities.

A linear relationship exists between pre- and post-conditions for atomic attacks and dynamic state variables. The computation’s difficulty is further influenced by the model’s size and the length of the attribute. Model size and security attribute length are known to have polynomial effects on complexity.

## 5. Conclusions

In this work, a novel method was proposed based on the application of SARSA RL to the attack graph comprising the set of possible attack scenarios performed against the system. The agent successfully found the best path that might cause the system the most harm. Our findings revealed which subsystem was most exposed to cyberattacks. The development of the best action selection guidelines to fix the vulnerabilities can be aided by these insights. Future improvements to this strategy might include the addition of the defender, who would take appropriate preventive action based on a constrained understanding of the state of the system, which would be made possible by the deployment of monitors. Additionally, the strategy can be illustrated more strongly on larger attack graphs. Moreover, alternative approaches, including deep RL and double RL, may be used and contrasted with the RL strategy that was employed in this research.

## Figures and Tables

**Table 2:** Reward matrix.

R	1	2	3	4	5	6	7	8	9
1	−1	3.4	4.7	−1	−1	−1	−1	−1	−1
2	0	−1	4.8	−1	−1	−1	−1	−1	−1
3	0	0	−1	8.1	8.1	8.7	8.7	10	10
4	−1	−1	0	−1	−1	−1	−1	−1	−1
5	−1	−1	0	−1	−1	−1	−1	−1	−1
6	−1	−1	0	−1	−1	−1	−1	10	10
7	−1	−1	0	−1	−1	−1	−1	10	10
8	−1	−1	0	−1	−1	0	0	−1	−1
9	−1	−1	0	−1	−1	0	0	−1	−1

## Data Availability

Not applicable.
